# Curcumin Attenuates Angiotensin II-Induced Abdominal Aortic Aneurysm by Inhibition of Inflammatory Response and ERK Signaling Pathways

**DOI:** 10.1155/2014/270930

**Published:** 2014-11-06

**Authors:** QingQing Hao, Xu Chen, XiaoYu Wang, Bo Dong, ChuanHua Yang

**Affiliations:** ^1^Department of Cardiology, Shandong Provincial Hospital Affiliated to Shandong University, Jinan, Shandong 250021, China; ^2^The Key Laboratory of Cardiovascular Remodeling and Function Research, Qilu Hospital, Shandong University, Jinan, Shandong 250012, China; ^3^Department of Pathophysiology, Fenyang College Shanxi Medical University, Fenyang, Shanxi 032200, China; ^4^Department of Cardiology, Affiliated Hospital of Shandong University of Traditional Chinese Medicine, Jinan 250011, China

## Abstract

*Background and Objectives*. Curcumin has long been used to treat age-related diseases, such as atherosclerosis and coronary heart disease. In this study, we explored the effects of curcumin on the development of abdominal aortic aneurysm (AAA). *Methods*. ApoE^−/−^ mice were randomly divided into 3 groups: AngII group, AngII + curcumin (AngII + Cur) group (100 mg/kg/d), and the control group. Miniosmotic pumps were implanted subcutaneously in ApoE^−/−^ mice to deliver AngII for 28 days. After 4-week treatment, abdominal aortas with AAA were obtained for H&E staining, immunohistochemistry, and Western blotting. *Results*. The results showed that curcumin treatment significantly decreased the occurrence of AAA. The levels of macrophage infiltration, monocyte chemoattractant protein-1 (MCP-1), and tumor necrosis factors-*α* (TNF-*α*) were significantly lower in AngII + Cur group than those in AngII group (all *P* < 0.01). The level of superoxide dismutase (SOD) was significantly higher in AngII + Cur group than those in AngII group (*P* < 0.01). The ERK1/2 phosphorylation in AngII + Cur group was significantly lower than that in AngII group (*P* < 0.01). *Conclusions*. These results suggested that curcumin can inhibit the AngII-induced AAA in ApoE^−/−^ mice, whose mechanisms include the curcumin anti-inflammation, antioxidative stress, and downregulation of ERK signaling pathway.

## 1. Introduction

Abdominal aortic aneurysm (AAA) is a severe disease threatening the human health and often causes death by rupture. The main pathological process of AAA includes (1) increasing inflammatory response, especially the macrophage infiltration and monocyte chemoattractant protein-1 (MCP-1) expression, (2) activating matrix metalloproteinases (MMP) and depredating extracellular matrix, and (3) degeneration of vascular media and damaging or breaking of elastic fiber [1, 2]. It is documented that statins, angiotensin receptor blockers (ARB), tissue inhibitors of metalloproteinases (TIMP), and some traditional Chinese medicines are beneficial for the treatment of AAA. However, no specific medicine can cure the disease. At present, the surgery is the only effective treatment method for AAA.

Recent studies have demonstrated that the renin-angiotensin system (RAS), especially angiotensin II (AngII), plays an important role in the pathogenesis of atherosclerosis and AAA. Lu et al. reported that subcutaneous infusion of AngII increases the size of atherosclerotic plaque and AAA formation in apoE-deficient mice (ApoE^−/−^ mice), suggesting that the imbalance of RAS is involved in the pathogenesis of AAA [3]. The AngII-induced aneurysm formation in ApoE^−/−^ mice mirrors many characteristics of the human AAA, such as increase of inflammatory response, enhancement of MMP activity, depredation of extracellular matrix, and rupture of aortic elastic layer. Furthermore, these studies showed that inhibition of RAS activation prevented AAA formation [3, 4]. Daugherty's group demonstrated that the AT1 receptor antagonist, losartan, prevented the formation of AAA and AT2 receptor antagonist, PD123319, enhanced the severity of AAA [1–4]. Another study reported that simvastatin prevented the AngII-induced AAA formation in ApoE^−/−^ mice by inhibiting MMP-2 and MMP-9 activity, MCP-1 protein expression, and ERK activity, indicating that inflammatory response and MAPK activation are involved in the pathogenesis of AAA [5].

*Curcuma* is the source of the spice, turmeric, which is widely used in Asian countries, especially in India and China. A number of scientific evidences have demonstrated that curcumin has many beneficial effects on health, including antioxidative and anti-inflammatory effects. Recent study has demonstrated that curcumin has an important role in prevention and treatment of age-related diseases, such as atherosclerosis, coronary heart disease, diabetes mellitus, and others owing to its powerful antioxidant and anti-inflammatory activities [6, 7]. The common characteristics of age-related disease are a low grade inflammatory response driven by oxygen stress and curcumin is believed to participate in age-related diseases by counteracting the inflammatory state and inhibiting oxygen stress production. Therefore, it has been widely used as a daily health supplement and treatment in cardiovascular disease. It was reported that curcumin protects the endothelial cell activity, inhibits vascular smooth muscle cell (VSMC) proliferation, and decreases the inflammatory response in vascular wall [8]. Quiles et al. found that* Curcuma longa* reduced oxidative stress production and prohibited the development of fatty streaks in aortic arch, thoracic aorta, and abdominal aorta in rabbits fed a high cholesterol diet [9].

Previous study found that curcumin could inhibit formation of rat aneurysm induced by calcium chloride method [10], but the result from other studies showed that calcium chloride-induced AAA did not reveal atherosclerotic plaque formation, vascular thrombus, and rupture, all of which represents classical features of human AAA [11].

The formation of aneurysm induced by the infusion of AngII has characteristic features that are much more similar to the human disease than other models of aneurysm induced by calcium chloride. The calcium chloride-induced AAA in rat only induces the formation of aneurysm, with no consideration of hyperlipidemia, gender, atherosclerosis, thrombus, rupture, and other complicated factors in human disease, but the aneurysm induced by AngII in ApoE^−/−^ mice generally considers atherosclerosis, thrombus, hyperlipidemia, gender, and rupture, and it is more fit for studying the mechanisms and treatment of AAA [1, 2, 11].

In this study, we established the AAA induced by AngII infusion and then observed the effect of curcumin on the AAA in ApoE^−/−^ mice. We hypothesized that curcumin prevents aneurysm development by inhibiting inflammatory response, MMP activity, and ERK signal pathway activation during AAA formation. We determined the effect of curcumin on the pathogenesis of AAA in ApoE^−/−^ mice. Our results showed that curcumin treatment significantly prevented AAA formation.

## 2. Materials and Methods

### 2.1. Ethics Statement

This study conforms to the principles outlined in the Declaration of Helsinki and to Chinese legal dispositions. This experiment was implemented in accordance with the recommendations in the guide for the care and use of laboratory animals published by the People's Republic of China (document number 55, 2001). All animal procedures were approved by the Committee on the Ethics of Animal Experiments of Shandong University.

### 2.2. Animal Modal

Male ApoE^−/−^ mice on a C57BL/6J background strain were housed under barrier conditions with food and water. Thirty-six mice (24-week-old male) were randomly divided into 3 groups (*n* = 12, each): AngII alone group (infusion of AngII, 1000 ng/kg/min, Sigma, St. Louis, MO, USA), AngII + Cur group (100 mg/kg/d, Sigma-Aldrich, St. Louis, MO, USA), and control group (infusion of normal saline). The mice were anaesthetized by intraperitoneal injection of ketamine (150 mg/kg) and xylazine (10 mg/kg). Miniosmotic pumps (Alzet Model 2004, Durect Corp, Cupertino, CA, USA) were implanted subcutaneously in mice to deliver AngII (1000 ng/kg/min) or saline for 28 days according to the literature as described previously [5, 8, 12]. Curcumin was dissolved in 1% carboxymethyl cellulose and was applied by daily oral gavage in AngII + Cur group. The curcumin treatment was initiated 1 week before AngII infusion and lasted for 28 days. At the end of experiment, the mice were anesthetized with xylazine 20 mg/kg and ketamine 100 mg/kg and the aortas were isolated for pathology examination.

### 2.3. Aortic Diameter Measurement and Aneurysms Diagnosis

Following anesthesia with xylazine 20 mg/kg and ketamine 100 mg/kg, the mice were cut open ventrally. Phosphate-buffered saline (PBS) was perfused into left ventricles and exited through the severed auricula dextra. Suprarenal region of abdominal aorta containing AAA was identified between the last pair of intercostal arteries and the right renal branch. After periadventitial tissues were removed from the aortic wall, the aortic wall was photographed and the maximal aortic diameter of the aorta was measured with a caliper. Aneurysms were defined and diagnosed by reference to the previous literature; the aneurysm diagnosis was made when the maximum diameter of the aorta exceeded 50% of the normal diameter.

The average diameter of normal suprarenal aorta in our control mice is ≈0.83 ± 0.04 mm. Therefore, we set a threshold of 1.25 mm as evidence of the incidence of aneurysm formation.

### 2.4. Specimen Preparation and Staining

The suprarenal region of abdominal aorta containing AAA was perfused with PBS and then either fixed in 10% buffered formalin solution or frozen in liquid nitrogen. For each mouse, one part of abdominal aorta containing AAA was used for histological examination and the other part was used for Western blotting and measurement of the oxidative radical.

The suprarenal aortas used for histological examination were harvested and dehydrated with different gradients of ethanol and then embedded with paraffin. Paraffin-embedded suprarenal aortas were cross-sectioned into pieces 4 *μ*m thick. H&E and Verhoeff Van-Gieson (VVG) staining were performed according to previous routine method.

### 2.5. Immunohistochemistry

The suprarenal region of abdominal aorta containing AAAs was serially cross-sectioned. The macrophages infiltration, MCP-1, TNF-*α*, MMP-2, and MMP-9 expression were analyzed by immunohistochemistry as described previously. The primary antibodies were used as follows: macrophages, MCP-1, TNF-*α*, and ERK1/2 were identified with a rabbit anti-mouse polyclonal antibody (Abcam, USA). MMP-2 and MMP-9 were detected with a monoclonal antibody (1 : 50, Abcam, USA). The cross-sections were labeled with primary antibodies at 4°C overnight. After washing, the bound antibodies were conjugated with secondary antibodies at 37°C for 1 hour, and then the DAB substrate was administrated and incubated for 1-2 minutes. The sections were counterstained with hematoxylin and the result was acquired with image analysis system (Image-Pro Plus 5.0, Media Cybernetics, USA).

### 2.6. RT-PCR

Total RNA was isolated from abdominal aortas containing AAAs using the TriZol method (Invitrogen, USA) according to the manufacturer's protocol. The concentration of total RNA was quantified by spectrophotometry and the mRNAs were reverse-transcribed into cDNAs using iScript cDNA synthesis kit (Bio-Rad, Hercules, CA). Quantitative real-time RT-PCR was performed with an Applied Lightcycler2.0 detection system (Roche Applied Science, USA) involving the SYBR-based method in 20 *μ*L reaction volume according to the manufacturer's instructions. The PCR primers of TNF-*α*, MCP-1, and *β*-actin are as follows: TNF-*α*, 5′-CCTGTAGCCCACGTCGTAGC-3′ and 5′-TTGACCTCAGCGCTGAGTTG-3′; MCP-1, 5′-ACTGAAGCCAGCTCTCTCTTCCTC-3′ and 5′-TTCCTTCTTGGGTCAGCACAGAC-3′; *β*-actin, 5′-TGCTGTCCCTGTAGTCCTCT-3′ and 5′-AGGTCTTTACGGATGTCAACG-3′. Reaction specificity was demonstrated by analyzing melting curves and by gel electrophoresis of the amplicons. The relative changes in gene expression were analyzed using a comparative method described in the applied biosystems user bulletin. The data were analyzed with 2^−ΔΔCT^ method.

### 2.7. Gelatin Zymography

The MMP-2 and MMP-9 activity were evaluated by methods of zymography according to previous literature [13]. Abdominal aortas containing AAAs were homogenized in a buffer containing 50 mM Tris/HCl (pH 7.5), 150 mM NaCl, and 1% Nonidet P-40. The homogenate was centrifuged and the protein concentration was measured by Bradford assay (Bio-Rad, Philadelphia, USA). 20 *μ*g of protein aliquots was used for each zymographic assay. The experiment of gelatine zymography was carried out under nonreducing conditions on 10% SDS-PAGE gel containing 0.1% (w/v) gelatin at 4°C. The gels were washed with a washing buffer containing 2.5% Triton X-100, incubated in reaction buffer (50 mM Tris-HCl, pH 7.4, 0.15 M NaCl, 5 mM CaCl_2_, 0.02% NaN_3_, and 0.05% Brij 35) at 37°C for 48 hours, and then stained with 2.5% Coomassie brilliant blue (Sigma Chemical Co., St. Louis, MO, USA). The band intensity was quantified by computer-assisted image analysis (Image-Pro Plus 5.0, Media Cybernetics, USA).

### 2.8. Measurements of SOD and MDA Assays

The SOD activity in the aneurysm was measured according to the method using a kit (NJBC, Nanjing, China). Tetrazolium salt can be made to form a red formazan dye by superoxide radicals generated by xanthine oxidase and hypoxanthine. The red formazan dye was measured and evaluated at the optical density at 550 nm by a spectrophotometer. The SOD activity was expressed as U/mg protein.

The MDA contents were measured according to thiobarbituric acid method. The procedure was carried out following the manufacturer's instruction (NJBC, Nanjing, China). The samples were determined at a wavelength of 546 nm using a spectrophotometer, and the results were expressed in terms of nmol/mg protein.

### 2.9. Western Blot 

ERK1/2 protein expression was detected by Western blot analysis. Mouse abdominal aortas containing AAAs were homogenized in a RIPA lysis buffer (Sigma Chemical Co.) containing protease inhibitors (Roche, Germany) and the total protein was extracted and detected. Twenty micrograms of each protein sample was electrophoresed on 14% SDS-PAGE. The protein was transferred onto a polyvinylidene difluoride (PVDF) membrane for 120 min at 250 mA. Following incubation in blocking solution, membranes were hybridized with 1 : 250 dilution of the primary anti-ERK1/2 antibodies (Santa Cruz Biotechnology, USA) overnight at 4°C. The membranes were then incubated with second antibodies for 60 min at room temperature. The visualization of immune-reactive bands was detected by enhanced chemiluminescence reagent. Quantification of the intensities of each band was performed by use of a MSF-300G Scanner (Microtek Lab, Nikon, Japan).

### 2.10. Serum Lipids and Blood Pressure Measurement

Serum cholesterol, low-density lipoprotein (LDL), and triglyceride (TG) level were measured by an enzymatic assay kit. The blood pressure was measured on mice using noninvasive tail-cuff systems (BP2010AUL, Softron) in the morning. Considering the procedure-induced anxiety, mice were acclimated to the blood pressure monitor and attemperator (TMC-213, Softron) for three days prior to these measurements. On the 27th day, six blood pressure values were taken on each mouse and averaged for analysis.

### 2.11. Statistical Analysis

Data analysis involved use of SPSS 11.5. Pearson's chi-squared test was performed to compare the incidence of aneurysm. One way analysis of variance was used to test the difference of means among three groups for continuous variables. A *P* value <0.05 was considered statistically significant.

## 3. Results

### 3.1. Curcumin Reduced the Incidence and Severity of AngII-Induced AAA Formation in ApoE^−/−^ Mice

**Figure 1:**
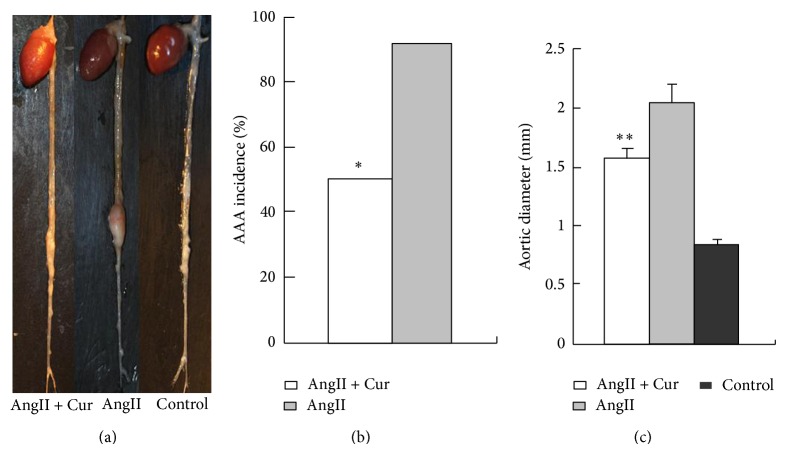
Curcumin reduces the incidence of AngII-induced AAA formation in ApoE^−/−^ mice. (a) Representative images of aortas aneurysm in three groups. (b) AAA incidencein two groups. ^*^ *P* < 0.01 versus AngII group. (c) Aortic diameter. ^**^ *P* < 0.001 versus AngII group or control group.

To evaluate the effect of curcumin on AngII-stimulated AAA formation, AngII-induced mice were treated with curcumin or vehicle. No aneurysms were present in saline-infused control ApoE^−/−^ mice (*n* = 12). In contrast, the result showed that eleven of 12 (or 92%) of AngII-induced mice developed aortic aneurysms (Figures 1(a) and 1(b)). However, this effect of AngII was statistically decreased in mice treated with curcumin; the result showed that 6 of 12 (or 50%) developed the aortic aneurysms, indicating that curcumin treatment significantly decreased the occurrence of AAA (Figure 1(b)).

There was no statistical difference in the incidence of aneurysm rupture between AngII + Cur group and AngII group (Table 1). The diameter of abdominal aortas in AngII alone group (2.04 ± 0.15 mm, *N* = 12) was significantly higher than that in control group (0.83 ± 0.04 mm, *N* = 12, *P* < 0.01). In contrast, the administration of curcumin treatment in AngII + Cur group (1.58 ± 0.09 mm, *N* = 12, *P* < 0.01) significantly decreased the aortic diameter compared to the AngII alone group (Figure 1(c)). The result demonstrated that curcumin treatment obviously attenuated the incidence and protected ApoE^−/−^ mice against AngII-induced AAA formation and development.

### 3.2. Curcumin Decreased Remodeling of Aortic Wall and Inhibited Inflammatory Response in AngII-Induced AAA

**Figure 2:**
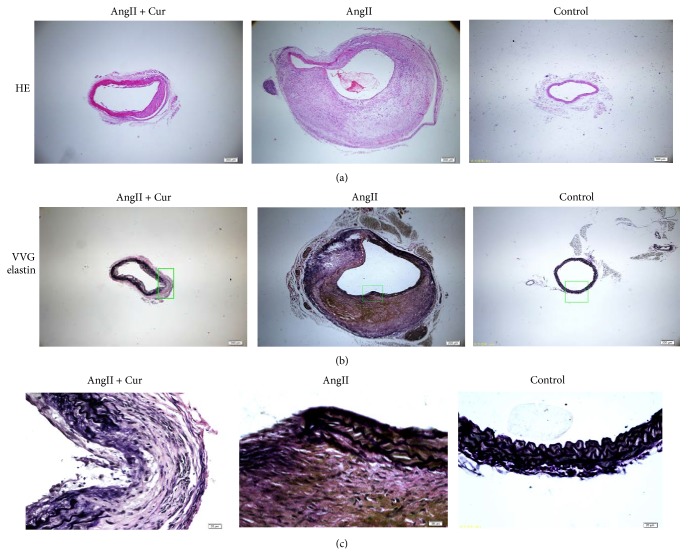
Curcumin prevents remodeling of the aortic wall. (a) The upper photos were H&E staining (40x, scale bars equal 200 *μ*m) of abdominal aortic cross-section from each group. (b) The middle photos were VVG elastin staining. Scale bars equal 200 *μ*m (40x, middle). (c) The bottom photos were the magnification of (b). Scale bars equal 20 *μ*m (400x, bottom).

H&E staining from the region of the aorta demonstrated that AngII treatment gives rise to a thickening of the abdominal aortic wall and disruption of the media and adventitia. VVG staining proved the disruption of the elastin fibers in the AngII alone group and AngII + Cur group (Figure 2). However, the adventitial thickness and disruption of the media and adventitia were relatively less marked in AngII + Cur group. There are no intimal thickening and the elastin fibers in control aortas are intact and not disrupted (Figure 2).

**Figure 3:**
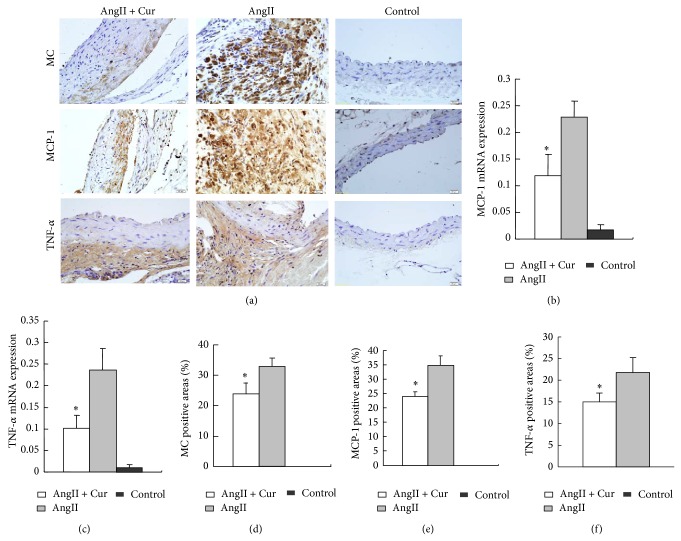
Macrophage, MCP-1, and TNF-*α* gene and protein expression by RT-PCR and immunostaining in three groups. (a) Immunostaining (scale bars equal 20 *μ*m, 400x) of abdominal aortic cross-section in three groups for macrophages (MC, macrophage) (top row), MCP-1 (second row), TNF-*α* (third row). ((b) and (c)) MCP-1 and TNF-*α* mRNA expression by real-time RT-PCR, ^*^ *P* < 0.01 versus AngII group. ((d), (e), and (f)) Quantification of the positive staining area of macrophages, MCP-1, and TNF-*α*. ^*^ *P* < 0.01 versus AngII group.

The result showed that AngII-infusion not only induced macrophage infiltration, but also increased MCP-1, TNF-*α* gene, and protein expression as evaluated by RT-PCR (Figures 3(b) and 3(c)) and immunohistochemical staining (Figures 3(a), 3(d), 3(e), and 3(f)). In contrast, the macrophage infiltration, MCP-1, TNF-*α* gene expression, and protein expression were significantly lower in AngII + Cur group than those in AngII alone group (all *P* < 0.01), indicating that curcumin treatment inhibited the inflammatory response in the pathogenesis of AAA.

### 3.3. Curcumin Inhibited MMP-2 and MMP-9 Expression and Activity

**Figure 4:**
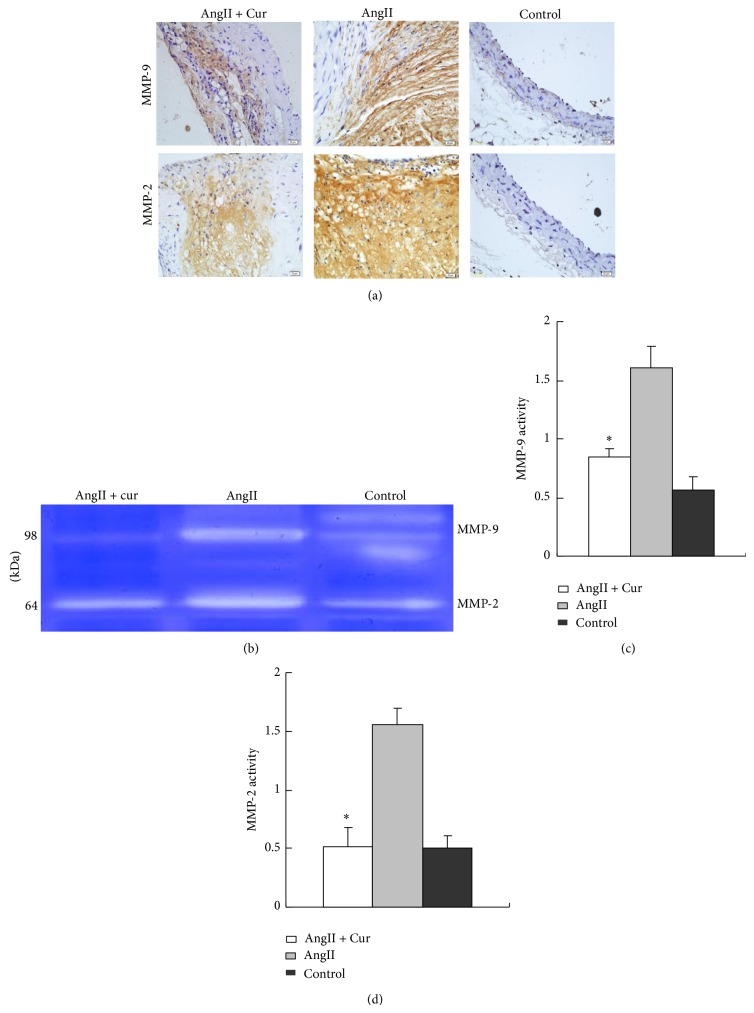
MMP-2 and MMP-9 protein expression and activity in three groups. (a) Immunostaining (scale bars equal 20 *μ*m, 400x) of abdominal aortic cross-section from AngII, AngII + Cur, and control groups for MMP-2 and MMP-9 protein expression. (b) MMP-2 and MMP-9 activity by zymography. (c) Quantification of the MMP-9 activity. (d) Quantification of the MMP-2 activity. ^*^ *P* < 0.01 versus AngII group.

The methods of immunohistochemistry and zymography were used to evaluate the MMP-2 and MMP-9 expression and MMP-2 and MMP-9 activity. The result showed that the staining of MMP-2 and MMP-9 by immunohistochemistry was extensive in AngII group as compared with that of AngII + Cur group (Figure 4(a)). The result also revealed that AngII-infusion in AngII group significantly increased the MMP-2 and MMP-9 activity by zymography (Figures 4(b)–4(d)). Conversely, curcumin treatment markedly inhibited MMP-2 and MMP-3 expression and activity in AngII + Cur group (all *P* < 0.01).

### 3.4. Curcumin Diminished ROS and ERK Signaling Pathways

**Figure 5:**
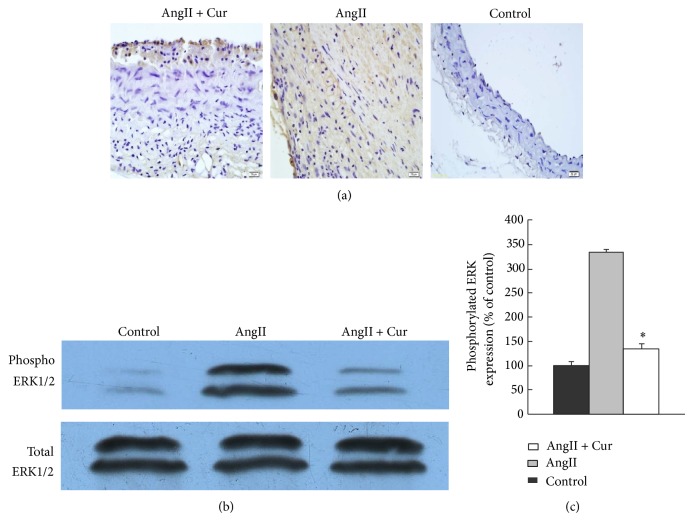
ERK protein expression by immunohistochemistry and Western blot. (a) ERK protein expression by immunohistochemistry in abdominal aortic cross-section (scale bars equal 20 *μ*m, 400x). (b) ERK protein expression by Western blot. (c) Quantitative analysis of results in (b). ^*^ *P* < 0.01 versus AngII alone group.

The result showed that the SOD was lower in AngII alone group compared to that of the control group. In contrast, the level of SOD increased in AngII + Cur group as compared with that of AngII alone group (*P* < 0.01). In addition, the level of MDA was increased in AngII alone group as compared with that in the control group. Furthermore, curcumin treatment reduced the MDA level, and the level of MDA was statistically lower in AngII + Cur group than that in AngII alone group (Table 2, *P* < 0.01). To explain the signaling pathways in aortic aneurysm, the ERK1/2 signaling pathways were evaluated. ERK1/2 phosphorylation in AAA of AngII alone group was significantly higher than that in AngII + Cur group (*P* < 0.01, Figures 5(b) and 5(c)); the result suggested that curcumin attenuated AAA pathogenesis at least by ERK1/2 signaling pathways.

### 3.5. Blood Pressure and Biological Measurements

Systolic blood pressure was measured in three groups, and the results showed that the systolic blood pressure was significantly higher in AngII alone group and AngII + Cur group than that in control group (*P* < 0.01), whereas systolic blood pressure was not statistically different between AngII alone group and AngII + Cur group. In addition, there was no significant difference in total cholesterol, LDL, and TG among AngII alone group, AngII + Cur group, and control group (Table 3).

## 4. Discussion

In the present study, we found that the incidence of AAA was significantly lower in AngII + Cur group than that in AngII alone group. Moreover, we demonstrated that the benefit of curcumin treatment on AAA is associated with the reduction of inflammatory response, decreased MMP-2 and MMP-9 activities, and inhibited ROS production. Furthermore, we reported that curcumin inhibits ERK phosphorylation in AAA.

RAS plays an important role in the pathogenesis of atherosclerosis and AAA. Previous studies have shown that AngII infusion induced AAA formation in ApoE^−/−^ mice. In line with the previous findings, our results demonstrated that AngII infusion increased the rates of AAA formation in ApoE^−/−^ mice. Thus, the AngII-induced AAA offered a good model to investigate the mechanism and pathogenesis of AAA in humans [5, 6, 14, 15].

Inflammation response and the local chronic inflammation of the aortic wall are characterized signs in the pathogenesis of AAAs [1]. Macrophage accumulation in the aortic wall is an early feature of AngII-induced AAAs and it presents in the whole process of AAA.

Our studies found that infusion of AngII induces marked inflammatory responses in the AAA which manifests increased macrophage infiltration and MCP-1 expression. However, curcumin administration inhibited MCP-1 expression and macrophage accumulation. In addition, curcumin also decreased TNF-*α* and IL-1 protein expression. Previous study also showed that curcumin decreased IL-1 and IL-6 level in C57Bl/6 mice induced AAAs with transient elastase perfusion [16]; the above result indicated that curcumin may prevent the formation of AAA by anti-inflammatory function.

It is well documented that MMPs play an essential role in the pathogenesis of AAA [17, 18]. MMPs are main enzymes that degrade the structural components of the ECM, and the formation and progression of AAA are accompanied by degradation of vascular ECM. Previous studies have shown that MMP activity is elevated in AAA. The level of circulating MMP-9 was significantly higher in patients with AAA than in those without AAA. Moreover, recent study reported that the level of MMP-9 in the plasma was statistically elevated in patients with ruptured AAA, compared to patients with nonruptured AAA [19]. Strikingly, it was reported that the inhibition of MMP activity prevented the development of AAA. Thus, the elevated MMP activity-induced vascular middle layer degradation is critical for the pathogenesis of AAA. In this study, we showed that AngII-infusion significantly increased the activity and expression level of MMP-2 and MMP-9. Conversely, curcumin treatmentsignificantly inhibited activity of MMP-2 and MMP-9. It has been previously shown that the activity of MMPs can be modulated by many inflammatory factors and oxygen radical [20, 21], so we speculated that mechanism of curcumin against MMP-2 and MMP-9 activities may be due to its anti-inflammatory and antioxidant effects. Taken together, these results suggest that one of the mechanisms of curcumin in preventing formation of AAA is by inhibition of activity of MMP-2 and MMP-9.

Increased oxidative stress is another important mechanism in the progression of AAA [22, 23]. It was reported that the production of oxidants is elevated and its antioxidants ability is decreased in AAA. Many studies have shown that curcumin has an important role in its elimination of reactive oxygen species. In this study, we found that the levels of superoxide dismutase (SOD) were significantly decreased, but the levels of malondialdehyde (MDA) were higher in the AngII group than those in AngII + Cur group; the result suggested that curcumin reduces oxidative stress production in AAA.

Oxidative stress can also influence the gene expression of signaling molecules such as MAPK and nuclear factor-*κ*B (NF-*κ*B). The previous study has shown that MAPK activation has been associated with macrophage infiltration in aneurysm and other inflammatory processes and with the activation of MMPs [5, 24]. Administration of CI1040, an inhibitor of ERK activation, attenuated the severity of aneurysm, macrophage infiltration, and MMP expression; the result has demonstrated that MAPK activation plays an important role in the pathogenesis of aneurysm [5]. Fan et al. showed that curcumin attenuates AAA formation by inhibiting JNK phosphorylation [10]. Zhang et al. found that the ERK was highly expressed in AngII-induced AAA formation. Another report found that simvastatin prevents AAA formation by inhibiting ERK phosphorylation [5]. It remains elusive whether curcumin treatment affects ERK phosphorylation in AngII-induced AAA. Our data showed that ERK phosphorylation significantly increased in the AngII group than that in the AngII + Cur group. Thus, our result indicated that curcumin reduces AAA formation at least in part via the inhibition of ERK signaling pathway. Previous study showed that nuclear factor-*κ*B (NF-*κ*B) signaling, the major transcription factor in mediating inflammation, can be activated in aneurysm. Katagiri's group established the mice model with endothelium transgenic expression of dominant-negative I*κ*B*α* (E-DNI*κ*B mice) and demonstrated that aneurysm formation is significantly inhibited in E-DNI*κ*B mice; the mechanism refers to inhibition of vascular inflammatory response and matrix metalloproteinases activation [25]. Study by Parodi et al. found that curcumin inhibits the NF-*κ*B activation and reduces the formation of aneurysm [16]. Previous study showed that curcumin attenuates cardiac inflammatory response, inhibits cardiomyocytic apoptosis, inhibits atherosclerosis, and prevents diabetic cardiomyopathy by inhibiting NF-*κ*B activity [26, 27]. From the above study, we speculated that the complex signaling pathway including MAPK and NF-*κ*B, the crosstalk between oxidative stress, and inflammation may be more important factor in the pathogenesis of aneurysms; the effect of curcumin on the treatment of aneurysm may be multiple-targeted including its anti-inflammatory and antioxidant effects by inhibition of multiple signaling pathways, including MAPK and NF-*κ*B signaling pathway.

In summary, our study demonstrated that curcumin significantly reduced the incidence of AngII-induced AAA formation in ApoE^−/−^ mice, and the protective effect of curcumin likely resulted from the reduced inflammatory response, decreased MMP-2 MMP-3 activity, and lowered ROS production. The mechanisms underlying these therapeutic effects involved downregulated ERK and AngII-ROS pathways. Thus, our study provides the new clue that daily use of curcumin is an effective approach to the prevention and treatment of AAA.

## Figures and Tables

**Table 1:** AAA formation and rupture in 3 groups of mice.

Groups	Control (*N* = 12)	AngII (*N* = 12)	AngII + Cur (*N* = 12)
AAA formation, *n* (%)	None	11 (92)	6 (50)^#^
AAA rupture, *n* (%)	None	8 (72.7)	4 (66.7)

^#^ *P* < 0.05 versus AngII or control group.

**Table 2:** The levels of SOD and MDA in 3 groups of mice.

Groups	SOD (U/mg protein)	MDA (nmol/mg protein)
AngII + Cur	100.73 ± 8.99^*^	3.05 ± 0.33^*^
AngII	74.79 ± 3.81	9.59 ± 0.61
Control	105.54 ± 10.36	1.90 ± 0.22

^*^ *P* < 0.01 versus AngII group.

**Table 3:** Effect of curcumin on serum total cholesterol (TC), triglyceride (TG), low-density lipoprotein (LDL), systolic blood pressure, and heart rate levels in 3 groups of mice.

Groups	TC (mg/dL)	LDL (mg/dL)	TG (mg/dL)	SBP (mmHg)	HR (beats/min)
AngII + Cur	600.37 ± 53.75	163.50 ± 8.67	105.79 ± 13.33	154.01 ± 6.47^*^	605.66 ± 35.12
AngII	603.73 ± 31.43	169.75 ± 4.43	112.84 ± 16.71	152.96 ± 4.92^*^	607.74 ± 38.27
Control	584.41 ± 52.14	159.25 ± 8.81	110.09 ± 16.66	112.39 ± 5.80	600.39 ± 15.10

^*^ *P* < 0.01 versus control group.
